# Short-Term Behavioural Responses of the Great Scallop *Pecten maximus* Exposed to the Toxic Alga *Alexandrium minutum* Measured by Accelerometry and Passive Acoustics

**DOI:** 10.1371/journal.pone.0160935

**Published:** 2016-08-10

**Authors:** Laura Coquereau, Aurélie Jolivet, Hélène Hégaret, Laurent Chauvaud

**Affiliations:** Université de Bretagne Occidentale, Institut Universitaire Européen de la Mer, Laboratoire des Sciences de l’Environnement Marin, UMR 6539, LIA BeBEST, Rue Dumont D’Urville, 29280, Plouzané, France; University of Connecticut, UNITED STATES

## Abstract

Harmful algal blooms produced by toxic dinoflagellates have increased worldwide, impacting human health, the environment, and fisheries. Due to their potential sensitivity (e.g., environmental changes), bivalves through their valve movements can be monitored to detect harmful algal blooms. Methods that measure valve activity require bivalve-attached sensors and usually connected cables to data transfers, leading to stress animals and limit the use to sessile species. As a non-intrusive and continuously deployable tool, passive acoustics could be an effective approach to detecting harmful algal blooms in real time based on animal sound production. This study aimed to detect reaction changes in the valve movements of adult *Pecten maximus* exposed to the toxic dinoflagellate *Alexandrium minutum* using both accelerometry and passive acoustic methods. Scallops were experimentally exposed to three ecologically relevant concentrations of *A*. *minutum* for 2 hours. The number of each type of valve movement and their sound intensity, opening duration, and valve-opening amplitude were measured. Four behaviours were identified: closures, expulsion, displacement, and swimming. The response of *P*. *maximus* to *A*. *minutum* occurred rapidly at a high concentration. The valve activity of *P*. *maximus* was different when exposed to high concentrations (500 000 cells L^-1^) of *A*. *minutum* compared to the non-toxic dinoflagellate *Heterocapsa triquetra*; the number of valve movements increased, especially closure and expulsion, which were detected acoustically. Thus, this study demonstrates the potential for acoustics and sound production changes in the detection of harmful algal blooms. However, field trials and longer duration experiments are required to provide further evidence for the use of acoustics as a monitoring tool in the natural environment where several factors may interfere with valve behaviours.

## Introduction

Over the last few decades, toxic marine dinoflagellate blooms have become more frequent worldwide, inducing dramatic human health and economic effects [1–3]. A number of dinoflagellate species, including those of the genus *Alexandrium*, produce paralytic shellfish poisoning (PSP) toxins, which can be an important source of marine toxins accumulated by bivalves. *Alexandrium minutum* is particularly well established in marine European coastal waters [1, 4]. Several areas in Brittany, France, are subjected to yearly intense blooms of *A*. *minutum*. The largest bloom caused by this species was documented in the bay of Brest in the summer of 2012, with more than 4 x 10^7^ cells L^-1^ [5–8]. This major bloom contaminated many commercial bivalve species, leading to the closure of several productive shellfish areas, including a fishery for the great scallop (*Pecten maximus*), which represents the third most exploited species in Brittany in terms of financial value and the fourth in terms of fishery volume.

Depending on the species, suspension-feeding bivalves do not have the same abilities to tolerate and accumulate paralytic toxins [9–11]. The behavioural and physiological responses of bivalves may be related to the toxicity of compounds produced by the algae [12], the concentration of toxins accumulated in bivalve tissues [13], and the history of harmful algal bloom exposure in the ecosystem [14]. Some bivalves that open and close their valves in response to changes in the environment could be a result of increasing or decreasing oxygen, heavy metals, salt, or harmful algal blooms [15–18]. Valve movements are closely related to vital activities, such as respiration, feeding, excretion, and moving. Changes in the patterns of valve activity in several bivalve species have been shown to indicate the presence of noxious dinoflagellates [14, 18–21]. Previous studies have highlighted the ability of *Alexandrium* blooms to impact the valve activity of oysters (*Crassostrea virginica* and *Crassostrea gigas*), quahogs (*Mercenaria mercenaria*), and mussels (*Perna viridis*) [18, 22, 23]. The behavioural responses of these species include changes in shell valve gap and valve microclosures. Monitoring such bivalve behaviours can prove information on the health of the shellfish and the water quality.

When exposed to *A*. *minutum*, the great scallop *P*. *maximus* is known to be able to change its physiological behaviours (e.g., rate of biodeposits, filtration, and absorption) [11, 24]. However, a precise description of the valve activity of this major commercial species in response to a harmful algal bloom has yet to be proposed. Measurements of valve activity require capturing and equipping organisms with measuring devices (i.e., valvometer or accelerometer [19, 25]), which can potentially affect their behaviour. Moreover, for real-time monitoring, these loggers usually require connecting cables, which limit this technique to sessile species. In the context of developing simple and non-intrusive marine monitoring tools, passive acoustics could potentially be a new approach to detecting harmful algal bloom events in real time. This method enables the collection of biological data over large spatio-temporal scales using hydrophones and has the valuable advantage of being applicable to mobile species and to not be limited in individual numbers. Some marine invertebrate species are sound-producers (incidentally or not), and can be recorded and studied [26]. Sounds play an important role in detecting early signs of animal stress [27, 28]. Unfortunately, sound production has been neglected in marine ecological investigations until recent years. Only few recent studies examined the effects of harmful algal blooms on marine biological sound production. Indeck et al (2015) [29] showed that ambient noise levels were significantly higher during the non-harmful algal bloom years due to an abundance of snapping shrimp sounds and fish chorusing, whereas Wall et al (2015) [30] did not detect any effect of red tide on fish sound production. However, no study of invertebrate sound production connected to harmful algal blooms has been conducted. Regarding *P*. *maximus* and previous acoustic studies, it has been shown that escape responses (swimming or displacement) and adduction movements produce specific sounds [26, 31]. The behaviours of this species have been described as being detectable in acoustic recordings and distinguishable from those of other known benthic organisms [31].

The present study evaluates if a coupled valve movement and acoustics analysis as a first step in the effort to assess whether an alert system could be developed using great scallops and passive acoustics. We described the short-term response of *P*. *maximus* valve activity after exposure to three ecologically relevant concentrations of *A*. *minutum* by characterizing, under laboratory conditions, behavioural changes that may indicate the immediate impact of *A*. *minutum* on the great scallop. To the best of our knowledge, this is the first study to attempt to record marine invertebrates exposed to toxic algae with passive acoustics.

## Materials and Methods

### Collection and maintenance of biological material

Adult *P*. *maximus* (n = 27, mean shell height ± standard deviation = 90.0 ± 4.8 mm) were caught with a scallop dredge in the Bay of Brest, France. In a context of study at the Institut Universitaire Européen de la Mer, no specific permission was required for the sampling in this study. We confirm that the sampling did not involve endangered or protected species. Scallops were transferred to a laboratory (chorus@lab, Fondation Grenoble INP) at the public aquarium Océanopolis in Brest. Epibionts were removed from the outer shell surface and the scallops acclimated to laboratory conditions 4 weeks before the start of the experimental sessions. The scallops were kept in three identical 120 L tanks (60 cm x 50 cm with a depth of 40 cm) for more space for each individual. The tanks were equipped for long-term housing and continuously supplied with fresh filtered seawater from the Bay of Brest (temperature: 13.0–13.5°C, salinity: 33.5–33.7). The animals were fed every 2 days with *Isochrysis* sp. (Tahitian strain) T-iso culture, a common aquaculture feed. All water parameters were controlled on a daily basis by the aquariology team of Océanopolis. Animals were maintained under a 11 h light/13 h dark photoperiod.

The AM89BM strain of *Alexandrium minutum* Halim (1960) isolated in 1989 in the Morlaix Bay in Brittany, France, and HT99PZ strain of *Heterocapsa triquetra* Ehrenberg (1840) (isolated in 1999 in the Penze estuary in Brittany, France, were used in these experiments. Cultures were grown in filter and autoclaved L1 medium [32] at 16°C with a 12:12 dark:light photoperiod (100 μmol photons m^−2^ s^−1^). Cultures were harvested at the end of the exponential growth phase. At this stage, the AM89BM strain has been reported to produce 1.3 ± 0.1 pg saxitoxin equivalents per cell [33]. *H*. *triquetra*, a nontoxic dinoflagellate commonly observed in the environment, was used as the control diet for the experiment. Microalgae concentrations were determined using a Nageotte cell under a light microscope.

### Measurement devices

#### Valve movement measurements

The AXY-2 data loggers (Technosmart) are accelerometers with the ability to record acceleration in three axes (range ± 4 ɡ, 39.24 m s^-2^) at 25 Hz with 8-bit resolution onto a 1 Gb RA memory card. The data loggers were heat-sealed into a polyethylene film. A piece of Velcro^®^ (1 cm^2^) was glued to the upper (left/flat) valve of each scallop and to a sealed data logger using glue (Araldite 90 s epoxy, Huntsman Advanced Materials). Including the memory card, the data loggers were a maximum size of 9.5 x 15 x 4 mm and had a mass under 2 g. The air inside the polyethylene film made the data logger neutrally buoyant in sea water.

#### Sound measurements

**Fig 1:**
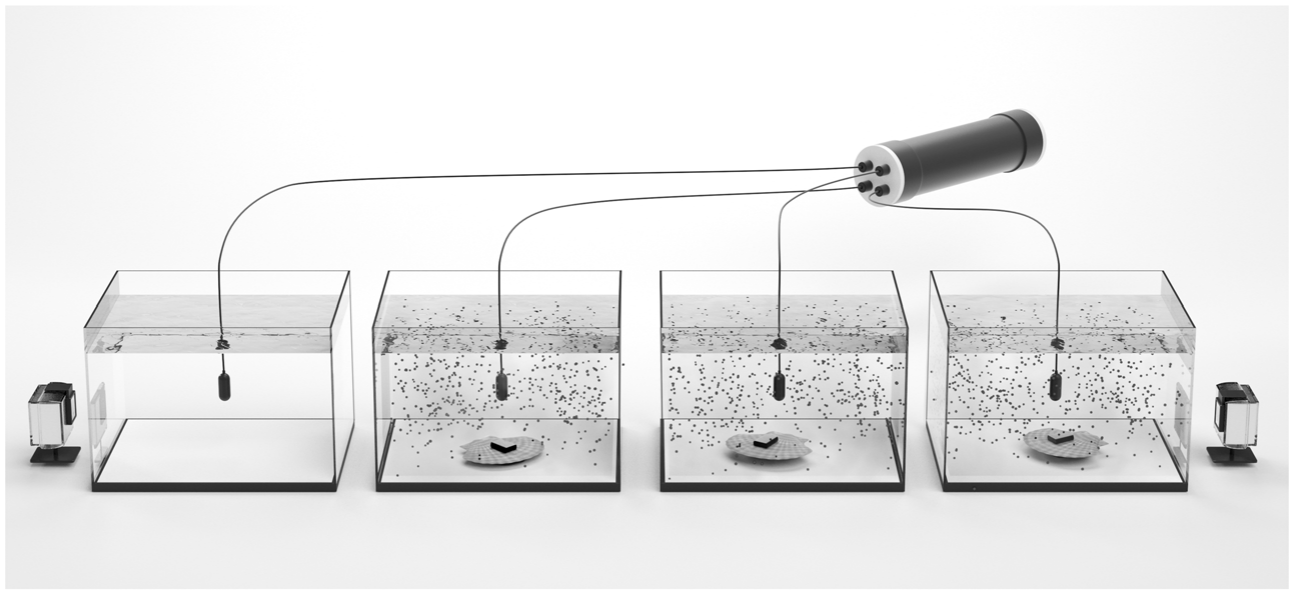
Experimental setup for recording valve movements and sounds of *Pecten maximus* under *Alexandrium minutum* exposures. Each scallop was equipped with an accelerometer on the upper valve. The hydrophones were suspended 15 cm above the bottom of each tank and linked to the acoustic recorder. A silicone plate (0.5 cm thick) on the bottom of the tank prevented sound emissions due to friction between hard body parts and the glass when animals moved. Black dots represent algae added in the tank at the beginning of the trial. Scallops were visually recorded using synchronized GoPro^®^ HERO 3 video cameras.

Acoustic recordings were acquired continuously using four HTI-92-WB hydrophones (High Tech Inc.), one in each experimental tank (Fig 1), with a sensitivity of -155 dB re 1 V/μPa and a flat frequency response over the range of 2 Hz to 50 kHz. The hydrophones were connected to an EA-SDA14 compact autonomous recorder (RTSys^®^). Acoustic recordings were acquired with a sampling rate of 156 kHz at 24-bit resolution. The hydrophones were suspended at the centre of the tank 13 cm above the silicone plate. To associate acoustic signal production and spikes from accelerometer data with behavioural events, scallop movements were followed using synchronized video cameras (GoPro^®^ HERO2) and/or visual observations.

### Experimental design

The experiment was performed in an isolated room with no human activity or extraneous noise to limit disturbance of the scallops. Three treatment groups, each with nine individuals, were fed different concentrations of algae cells: 5 000 cells L^-1^, corresponding to a relatively low concentration of *A*. *minutum* bloom, which can be regularly observed in the Bay of Brest; 10 000 cells L^-1^, corresponding to the sanitary alert threshold of *A*. *minutum* concentration defined by the French monitoring network REPHY in France; and 500 000 cells L^-1^, corresponding to the high concentration of *A*. *minutum* observed regularly in several areas of the Bay of Brest since 2012. These concentrations were also environmentally relevant for *H*. *triquetra*, which is one of the most common bloom-forming dinoflagellates found in estuaries and near shore regions around the world [34, 35]. Each replicate experiment was conducted during the light phase (08:00–19:00) and took place over two following days. On the first day of the experiment, each group was fed the control diet (*H*. *triquetra*) at the specified concentration. On the second day, the groups were fed the toxic diet (*A*. *minutum*). Each individual was assigned to a control treatment on the first day and then assigned to the matching toxic treatment on the second day. For each individual, control and treatment recording sessions were run as much as possible at the same period of the day (± 1 h) between the two days.

Experiments were performed using four identical 100 L tanks (50 cm x 50 cm x 40 cm, Fig 1). Three of them were used to run experiments with scallops. One, without scallop, nor alga, served as an acoustic control to record ambient noise and validates detected sounds from the three other tanks. The bottom of each tank was equipped with a silicone plate (0.5 cm thick) to prevent sound emissions due to the friction of shells on the glass when the animals moved. Before each trial, the tanks were carefully washed to remove microalgae from the previous trial and filled with fresh filtered seawater. During the recording sessions, there was no seawater exchange in the tanks in order to avoid additional noise.

Trials were performed separately for each individual. Animals were removed from their acclimation tank, transferred to the centre of one of the three experimental tanks, and a custom-made accelerometer (Technosmart, Italy) was attached with the piece of Velcro^®^ on the upper valve (Fig 1). After an acclimation period of 30 minutes in the experimental tanks, which was sufficient for scallops to show a typical valve gape and deploy their tentacles, microalgae were added to the three tanks and the water mixed gently with a glass rod to homogenize the microalgae concentration. After approximately 1 minute of homogenization, sound was recorded for 2 hours, which corresponds to the time for observing a thin algal layer on the silicone plate as a result of algal sedimentation since the sea water exchange was turned off.

### Data analysis

#### Accelerometer data

Data from the accelerometers were downloaded onto a PC using AxyManager software (Technosmart). Acceleration was given in units of ɡ, where ɡ represents acceleration due to gravity (1 ɡ = 9.81 m s^−2^). The x-axis of the data logger measured sway, the y-axis surge, and the z-axis heave [36]. The acceleration data were processed using the FullDBA and BeFeatures packages within BEnergetix in the free R software [25]. Briefly, an approximation of absolute acceleration resulting only from dynamic acceleration in each of the three dimensions was extracted from each axis after removing the static acceleration using a running mean of 3 s [36]. A derivative of dynamic body acceleration, the vector sum of dynamic body acceleration (VeDBA), was calculated from tri-axial acceleration data as VeDBA = √ (ax2 + ay2 + az2). Here, ax, ay, and az are dynamic acceleration values derived from raw x, y, and z acceleration data [37]. Gravitational acceleration can also be used to determine the orientation of a body in space (body angle), calculating the body “pitch” angle from the heave or surge axes and body roll from the sway axis [25].

As the accelerometer is glued to the upper (left/flat) valve of the scallop, pitch angle variations were used as a proxy of the angle of shell opening/closing and roll angle variations as a proxy of shell movement or rotation. For example, when the shell is closed and placed on the bottom, the pitch angle is 90°. When the shell opens, the lowest valve does not move and the pitch angle decreases in proportion to the opening angle of the shell. Each closure corresponds to the pitch angle returning to the reference value (i.e., the value obtained when the shell is closed).

#### Acoustic analysis

Sound recordings (.wav files) were processed using Raven Pro 1.5© (Cornell Lab of Ornithology) and specific signal processing routines developed in Matlab^®^. The quality of the recordings in terms of absence of interfering sounds was checked. The sound data were analysed in terms of the number of visible higher energy events compared to the surrounding background noise. Sound signals were detected manually. From these signals, we extracted two sound features following the methods described in [31]: received level (RL; in dB re 1 μPa root mean squared (rms)) and duration (T; s). The RL, which corresponded to the sound intensity, was calculated in the time window equal to signal selection, and duration was obtained along the frequency and time axes of the set of pixels comprising the signal selection. Mean and standard error (SE) values were determined for the acoustic features of each behaviour type and algae treatment. Signals were assigned in groups according to their shape and acoustic features, then these groups were validated with GoPro footages/observations and accelerometer data. Small glass-sided tanks create a highly reverberant and complex acoustic environment, resulting in distorted acoustic features [38–40]. Thus, the raw values of these features do not represent exactly sounds that would be recorded under field conditions. In this context, we recommend using these values in relative terms to compare sound production between behaviour types. Future work can then establish the precise acoustic values of valve movements in natural conditions.

To identify the relationship between accelerometer and acoustic data, recordings were played simultaneously. These data were also compared with visual observations or images taken by cameras, which were recorded simultaneously.

#### Statistical analysis

Results are expressed as mean ± SE. Differences between the two algal diets were investigated using the non-parametric Wilcoxon signed rank test for each concentration (for example, high concentration of toxic algae vs high concentration of control algae for the total number of movements) because assumptions of normality of data and equal variance tests were not validated. To compare the effect of the concentration (for example comparison between high vs medium vs low concentrations of toxic algae for the total number of movements), a Welch’s ANOVA test was applied. For all statistical results, *p* < 0.05 was considered significant. All analyses were performed with R Studio 3.0.2© software.

## Results

### Detection of scallop valve movements

#### Valve movement recordings using accelerometers

**Fig 2:**
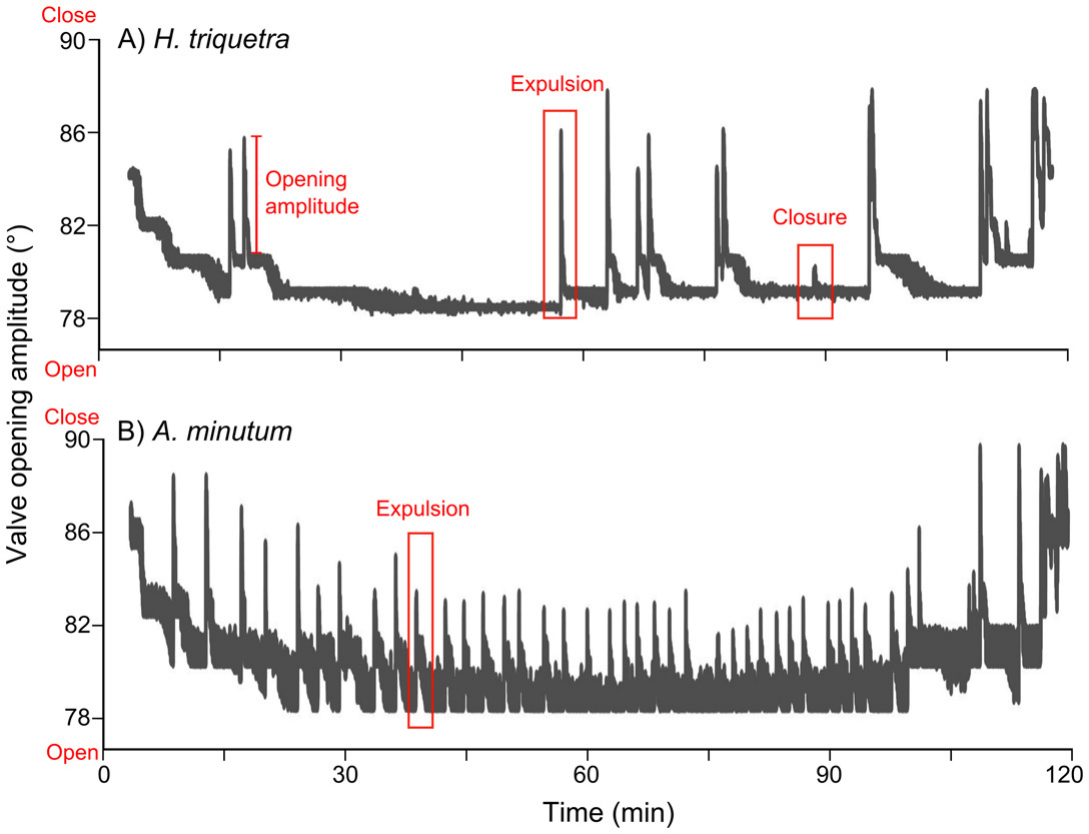
Valve movement recorded by accelerometry. Data obtained from a single great scallop exposed for 2 hours to the higher cell concentration (500 000 cells L^-1^) of A) *Heterocapsa triquetra* control treatment and B) the toxic *Alexandrium minutum*.

During the 2 hours of exposure, scallops opened their valves and appeared to filter actively under both control and toxic diets. For both diets at all concentrations, the accelerometer data showed that the continuous recordings of the scallop valve movements (Fig 2) were marked by long periods with a flat line (i.e., shell without movement) interspersed with more or less high pulses (i.e., shell closure of short duration). During the recording sessions, four behaviour types were identified (Fig 2): displacement was the result of an increase in the opening amplitude before rapid shell closure inducing shell displacement (variation in pitch and roll angles > 3°); swimming was a succession of shell displacement events associated with water expulsion, with the valve reopening quickly between flapping (time between displacement events < 1 second); expulsion was a rapid valve adduction associated with the expulsion of faeces, water, and other substances from the mantle cavity but not sufficient to displace the shell (variation in pitch angle > 3° and variation in roll angle < 3°); and closures were observed as minor contraction movements to return to an optimal opening amplitude (variation in pitch and roll angles < 3°). Accelerometer data always matched with video footages. For all exposure conditions, the order of behaviour type abundances were the same: closure was the most common behaviour (53 ± 2% of the total number of movements), followed by expulsion (25 ± 3%), displacement (19 ± 3%), and swimming (3 ± 1%).

#### Acoustic data validation

**Fig 3:**
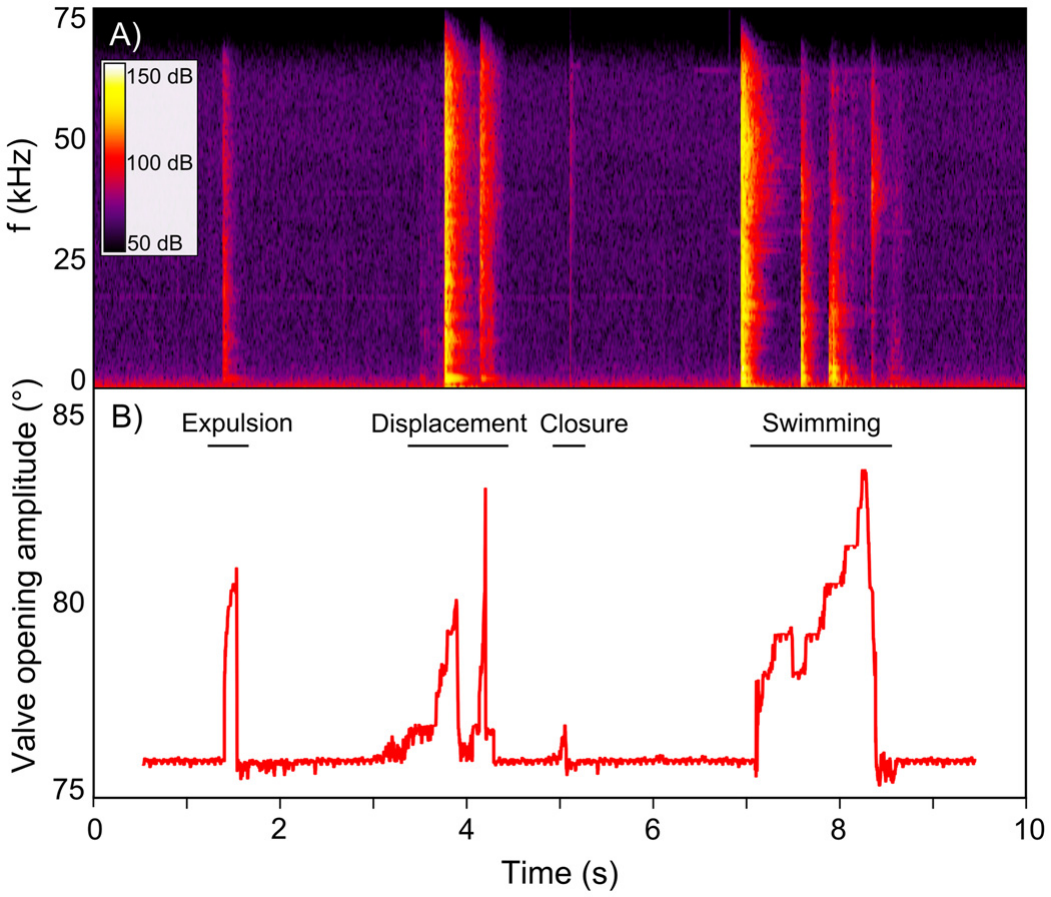
Accelerometer *vs*. acoustics methods. Comparison of *Pecten maximus* movements (expulsion, displacement, closure, and swimming) obtained with A) passive acoustics and B) accelerometry.

Acoustic signals were identified by careful visual inspection of the spectrograms of acoustic recordings and distinguished as short impulsive events of higher energy compared to the surrounding background noise. Analyses of acoustic recordings taken in the empty control tank confirmed the absence of any significant acoustic signal outside the experimental tanks during each sessions and the sound transfer from scallop movement from a tank to another. Signals from the scallops could be separated in four groups according to their general shape, sound intensity and duration. These groups were validated with both video and accelerometer recordings. Fig 3 shows a representative visualization of typical scallop valve movement types (expulsion, displacement, closure, and swimming) simultaneously obtained from acoustic (Fig 3A) and accelerometer (Fig 3B) recordings. Low frequencies (<1 kHz, Fig 3A) exhibited a continuously high energy level, which could be attributed to the acoustic recorder intern sound, whereas valve movements were characterized by a high-energy band in the vertical plane between 2 kHz and 75 kHz (Fig 3A).

The four valve movement types were represented by short spikes and perceived by the human ears as shorts cracks. When comparing accelerometer and acoustic data, we noted that a total of 45% of valve movements were detected in acoustic recordings. Visual detection of event number on the spectrogram was higher for swimming and displacement than for expulsion and closure. Indeed, a comparison of both accelerometer and acoustic data showed that 100% of swimming and displacement events were detectable in acoustic recordings, but only 51% of expulsion and 18% of closure events.

Acoustic features varied between the different valve movement types. Swimming and displacement events had a similar mean sound intensity (95.0 ± 2.5 dB re 1 μPa *vs*. 94.5 ± 2.5 dB re 1 μPa, respectively), as did expulsion and closure events (80.5 ± 1.7 dB re 1 μPa *vs*. 81.5 ± 1.9 dB re 1 μPa, respectively). Thus, the two last behaviours appeared to be 25% less intense than swimming and displacement. Sound duration varied between behaviours: swimming lasted the longest at approximately 2.5 ± 0.8 s, followed by displacement (0.73 ± 0.1 s), expulsion (0.1 ± 0.02 s), and closure (0.01 ± 0.005 s).

### Scallop behaviour under control *versus* toxic exposure

**Fig 4:**
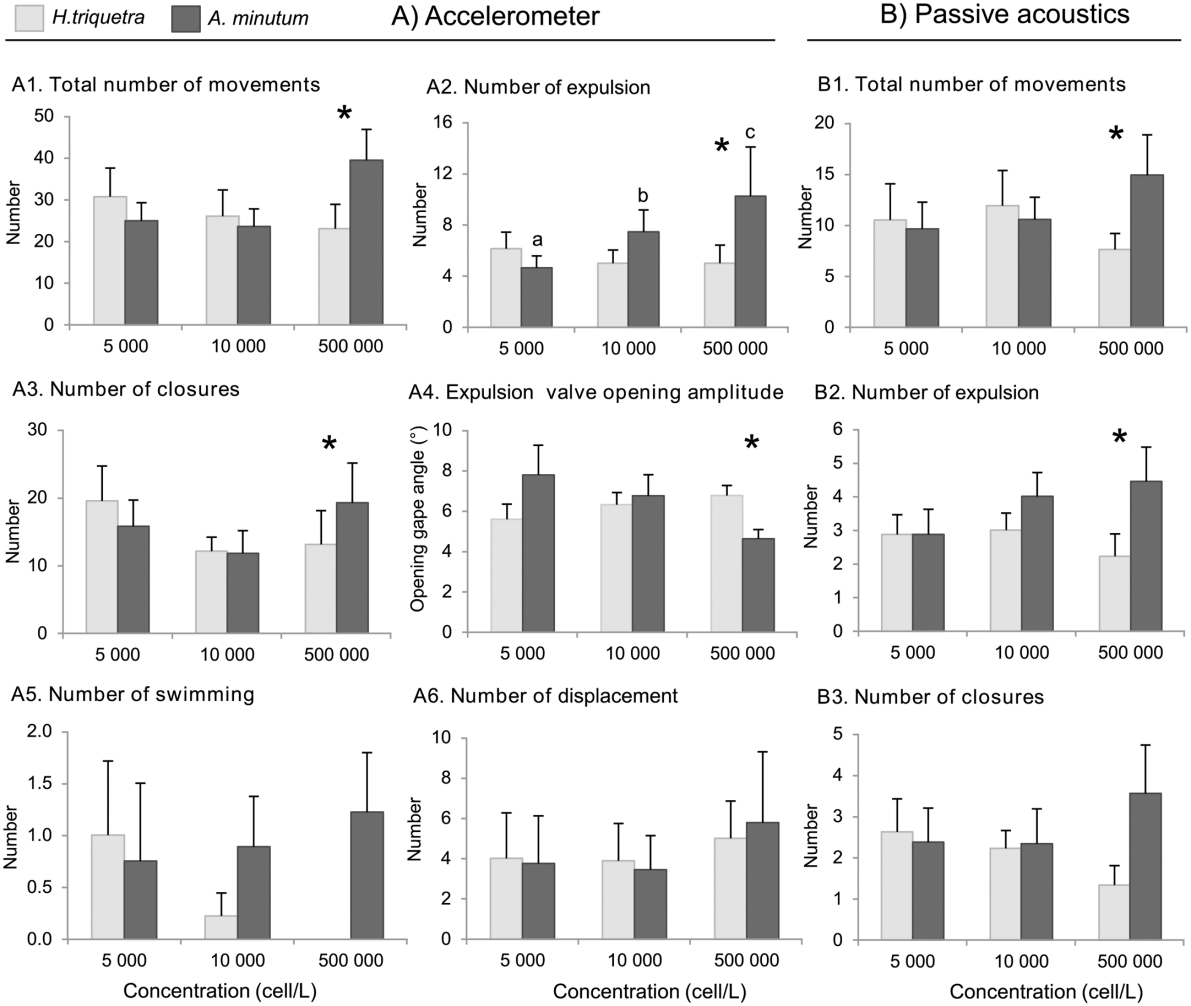
Effects of *Alexandrium minutum* on behaviour. A) Accelerometer and B) acoustic data showing movement responses in *Pecten maximus* exposed to three concentrations of *Heterocapsa triquetra* (light grey bars, control) or toxic *Alexandrium minutum* (dark grey bars) for 2 hours (mean ± SE, n = 9 per concentration). Asterisks indicate significant differences (Wilcoxon signed rank test) between toxic and control diets for each concentration. Different letters indicate significant differences (Welch’s ANOVA) between the three concentrations of a diet. The graphs describing the number of swimming and displacement detected by passive acoustics were not presented since these behaviors were always detected with acoustics, and thus correspond exactly to those obtained with accelerometer.

Analyses of the number of movements, the number of expulsions, and the number of closures exhibited by scallops over different time intervals (10 min, 15 min, 30 min) over the two hour period showed a stable activity over time in the presence of both algae at the three concentrations (S1 Fig). Exposures to 5 000 cells L^-1^ and 10 000 cells L^-1^ *A*. *minutum* resulted in similar valve movement patterns as *H*. *triquetra* exposure at the same concentrations, but significant differences were obtained between the two algal treatments at 500 000 cells L^-1^ (Fig 4). These results were given by both accelerometer and acoustic data.

When exposed to a concentration of 500 000 cells L^-1^, both methods detected a significant (by a factor of 2) increase in the number of movements in the presence of *A*. *minutum* compared to the control diet *H*. *triquetra* (*p* = 0.034 and *p* = 0.021 for accelerometer and acoustic, Fig 4A1 and 4B1). Accelerometry data showed that the number of expulsion and closure events increased by a factor of 2 in the presence of *A*. *minutum* compare to the presence of *H*. *triquetra* (Wilcoxon tests, *p* = 0.029 and *p* = 0.039, Fig 4A2 and 4A3). The number of expulsion events detected by acoustics also significantly increased by a factor of 2 when exposed to *A*. *minutum* at 500 000 cells L^-1^ compared to the control diet *H*. *triquetra* (Wilcoxon test, *p* = 0.013, Fig 4B2). However, despite not detecting a significant difference in the number of closures that could be detected by acoustic recordings (Wilcoxon test, *p* = 0.057, Fig 4B3), a clear tendency was observed. No significant effect was detected for swimming and displacement behaviors with both accelerometry and acoustic methods, even at high concentration (Wilcoxon tests, *p* > 0.05, Fig 4A5 and 4B6). Accelerometer data demonstrated that the expulsion number significantly increased with increasing *A*. *minutum* concentrations (Welch’s ANOVA test, *p* = 0.003, Fig 4A2). However, this significant increase was not observed with acoustic data (Welch’s ANOVA test, *p* = 0.58, Fig 4B2).

Finally, accelerometer data showed that exposure to *A*. *minutum* caused changes in the opening amplitude of expulsion compare to exposure of *H*. *triquetra* at 500 000 cells L^−1^ (Wilcoxon test, *p* = 0.03, Fig 4A4). However, no difference in the mean opening amplitude (resting periods) was detected during the 2 hours of exposure, which was 3° ± 0.2 and 3.5° ± 0.4 for all trials.

No other valve movement patterns, such as acceleration, mean duration, or mean valve gape for a movement type, were affected by exposure to the toxic treatment compare to the control treatment at either concentration (Wilcoxon tests, *p* > 0.05). The total number of movements and the number of expulsion, displacement, and closure events were stable during the 2 hours for all exposure conditions. Acoustically, no difference was detected in intensity or event duration for the different behaviours between the two algal diets.

## Discussion

This study demonstrates that, under laboratory conditions, short-term exposure of *P*. *maximus* to the bloom concentration of toxic dinoflagellate *A*. *minutum* affects some aspects of its behavioural response, which can be detected by both accelerometry and acoustic methods. This study is also the first attempt to establish the usefulness of passive acoustics as an innovative and non-invasive tool for monitoring the occurrence of harmful algal blooms based on bivalve behaviour and changes in sound production.

During the exposure of *P*. *maximus* to *H*. *triquetra* (the control diet) and *A*. *minutum* (the toxic diet), the scallops remained open nearly all the time, which seemed to ensure continuous ventilation and constant inflow through the mantle cavity, interrupted by short closures and other movements, such as expulsion, displacement, or swimming. These four movement types were detected and characterized by accelerometry and acoustics, demonstrating its potential for monitoring scallop valve movements.

For all exposure conditions, the order of behaviour type abundances were the same: closure > expulsion > displacement > swimming. At low concentrations (5 000 and 10 000 cells L^-1^), no significant effect was detected on the valve behaviour of *P*. *maximus* compared to the control exposures during the 2 hours of exposure. However, when *A*. *minutum* was present at a concentration of 500 000 cells L^-1^, the bloom concentration recurrently observed in Brittany and especially in the Bay of Brest [5, 6], accelerometry and acoustics showed that scallops significantly increased their total number of valve movements by a factor of 2, compared when exposed to the control diet. This was indicated by an increase in closures and expulsion by a factor of about 2. Similar increases in valve activity following exposure to toxic dinoflagellates have been described in oysters and clams [14, 16, 18–21, 23, 41]. Tran et al. [18] suggested that the biological significance of changes in these valve activities upon *A*. *minutum* exposure could be due to an avoidance response after contact between *Alexandrium* cells and external organs, a protective behaviour to avoid contamination or as a response to the toxins excreted by the cells. Furthermore, the increased movements detected in this study (closures and expulsion) may serve as a reaction to reject invasion by *A*. *minutum* cells from the mantle cavity, clean the gills, or refresh the water flowing inside the animal, especially expulsion, which allows the expulsion of captured material [42]. Even though *P*. *maximus* is a mobile species, individuals did not try to escape as a direct response to the presence of *A*. *minutum*, as the numbers of displacement and swimming events were not different from the controls. On the other hand, when exposed to *Alexandrium* sp., several bivalve species, including oysters, clams, and mussels, react with a decrease in valve gape [18, 22]. In our study, the valve gape of expulsion was significantly different between the two algal treatments at the higher concentration. However, the valve gape outside active periods was identical in all exposure conditions. These observations suggest that *P*. *maximus* keep a stable valve gape to filter even in the presence of the toxic algae but reacts, and may protect itself, during movements that require an increased valve gape in order to, for example, expel pseudofaeces more efficiently. A 2-days control (i.e. day 1: scallops fed with *H*. *triquetra*, day 2: scallops fed with *H*. *triquetra*) would control for possible time effect between the two diet treatments. Furthermore, additional studies should be developed to better characterize the impact of *A*. *minutum* on scallop valve responses. It would be interesting to investigate the impact of *A*. *minutum* on the valve responses of scallops over a longer period of exposure, to better evaluate this absence of effect at low-medium concentrations and possibly detect an impact after a few hours of exposure. Assessing the threshold concentration at which significant changes can be observed would also be valuable.

A major challenge identified in the possible use of scallop sounds to detect environmental changes, such as the presence of *A*. *minutum* bloom, was to distinguish whether the changes resulted in a significant difference in detected acoustic signals compared to “normal” conditions. This study highlighted that both swimming and displacement events were always detectable in acoustic recordings, unlike closures and expulsions that were the behaviors significantly different from the control at high concentrations. Several parameters, such as the maximum opening amplitude during the movement, the rotation of the shell, and the proximity or position of the scallop next to the hydrophone, could explain why closure and expulsion were not always detected. However, no correspondence could be found between any of these parameters and the event detection. In regards to closure, the non-detection by acoustics can be explained by the low sound intensity associated with a very short duration, as it was probably confounded by the background noise. The velocity and quantity of expelled water during valve movement, which is known to be variable [43], could play a role in the acoustic detection of these behaviours, especially expulsion. Feature combination analyses of signal shape, sound intensity, and duration, would result to a reliable distinction between the four behavior types in the field where no visual validation is possible.

At the concentration of 500 000 cells L^-1^, the total number of valve movements and the number of expulsion events were acoustically significantly different from controls. Thus, despite the fact that closure and expulsion behaviors were the quietest identified with passive acoustics, a significant increase in the number of events was observed with acoustics after *A*. *minutum* exposure. Di Iorio et al. [31] recorded *P*. *maximus* valve movements to evaluate the potential use of these sounds in monitoring environmental changes. These authors demonstrated that expulsions (called coughs) can be monitored *in situ* using passive acoustics and can be detected from a few to several tens of metres in different ambient noise environments, especially in the Bay of Brest. Furthermore, they showed that the acoustic characteristics of scallop expulsion are distinctive from any sound produced by other known benthic organisms, indicating that this behaviour is potentially interesting as indicator of changes in the environment.

In a possible context of monitoring harmful algal blooms with passive acoustics method, the analysis of both the total number of valve movements and the number of expulsions would result in a stronger comparison before, during and after the bloom. They clearly appeared as potential parameters to follow in order to characterize the presence of an *Alexandrium* sp bloom. It could be conceived to house a certain number of scallops under large cages with hydrophones above to perceive a great number of signals, including the quietest. Further experiments, with longer exposure periods would also be necessary in order to confirm the results of the effect of *A*. *minutum* on swimming and displacement behaviors. Indeed, these behaviors being always detected acoustically would even reinforce passive acoustics usefulness in a context of harmful toxic algal bloom.

Passive acoustics presents many advantages compared to accelerometry, as it results in no stress for the animal due to handling and it can be performed on a large number of individuals and on mobile species. Moreover, the acoustic characteristics of marine sites were previously reported to correlate with ecological properties and to be useful for monitoring environmental changes [44–48]. Thus, acoustic methods appear to be a potential non-intrusive tool to rapidly assess ecosystem health over relevant spatiotemporal scales and to identify biodiversity changes in marine environments, where direct visual observation is often not feasible. Previous studies have shown that bivalve responses to *A*. *minutum* are different than behavioural responses to trace metals, which they react to by closing their shells [49–51], to oxygen deficit, to which they increase the duration of valve gape [52], or to temperature variations [53]. Thus far, no data are available for acoustic changes in *P*. *maximus* valve movement in the presence of environmental variations or stressors. The specificity of these valve movements and sound responses to harmful algal blooms remains to be assessed, but this study clearly highlights the potential for passive acoustics, especially expulsion, as a potential tool for monitoring the presence of *A*. *minutum* in the natural environment. Importantly, sound interference may be caused by boats or water conditions (rushing currents, waves, high winds), which makes passive acoustics somewhat more difficult to use offshore. However, these disadvantages can be minimized if the monitoring analyses are investigated on the nigh part recording and under favourable sea conditions. Harmful algal blooms often begin in spring when winter storms are over and the sea calmer. The alternative of passive acoustic survey could be an efficient system, offering opportunities for monitoring several and mobile species simultaneously, excluding or reducing human intrusion in the surveyed area. It is necessary to complement these preliminary results with field experiments to provide evidence for the use of acoustic tools in the natural environment, where several factors may interfere with valve behaviours.

## Conclusions

Changes in the valve behaviours of *P*. *maximus* under short-term *A*. *minutum* exposure in the laboratory suggested that this bivalve species is sensitive to the alga at high concentrations and could be valuable as an indicator species for the early detection of harmful algal blooms. The sanitary threshold defined by the French harmful algal monitoring network REPHY developed by IFREMER is 10 000 cells L^-1^. Thus far, accelerometry and passive acoustics do not seem to allow the detection of such concentrations in the water, but this approach should be continued to define the behavioural responses of *P*. *maximus* to *A*. *minutum* over a longer period of time and assess whether a response can be detected over time at a concentration of 10 000 cells L^-1^. It would also be very interesting and valuable to assess the threshold concentration above which significant responses can be detected with passive acoustics. Even though specific experiments in the field are still required to test the acoustic assumptions, the present work clearly highlights that changes in the acoustics of *P*. *maximus* valve movement have the potential to be utilized in a powerful, non-intrusive method of monitoring the occurrence of harmful algal blooms.

## Supporting Information

S1 Fig Activity stability over the 2 hours recording. Scallop valve activity over the 2 hours of recording in response to exposition of *Heterocapsa triquetra* or toxic *Alexandrium minutum*. The examples here are under concentration expositions of 500 000 cell/L.
(PDF)

S1 File Video of scallop behaviours. Video presenting the four valve behaviours recorded in this study.
(MP4)

S1 Table Number of detected movements. Summary of detected movement number found by both accelerometer and acoustics methods for each recording.
(PDF)
