# Supplementary material for: Short-Term Behavioural Responses of the Great Scallop Pecten maximus Exposed to the Toxic Alga Alexandrium minutum Measured by Accelerometry and Passive Acoustics
Source: PLoS One. 2016 Aug 10;11(8):e0160935. doi: 10.1371/journal.pone.0160935 (PMC4980006; doi:10.1371/journal.pone.0160935)

Short-term behavioural responses of the great scallop *Pecten maximu* s exposed to the toxic alga *Alexandrium minutum* measured by accelerometry and passive acoustics

Laura Coquereau [a,*], Aurélie Jolivet [a,b], Hélène Hégaret [a], Laurent Chauvaud [a]

[a] *Université de Bretagne Occidentale, Institut Universitaire Européen de la Mer, Rue Dumont D'Urville, 29280 Plouzané, France*
[b] *TBM environnement/Somme, 115 rue Claude Chappe, Technopole Brest Iroise, F-29280 Plouzané, France*

Figure 1 Scallop valve activity over the 2 hours of recordings in response to exposition of *Heterocapsa triquetra* or toxic *Alexandrium minutum.* The examples here are under concentration expositions of 500 000 cell/L

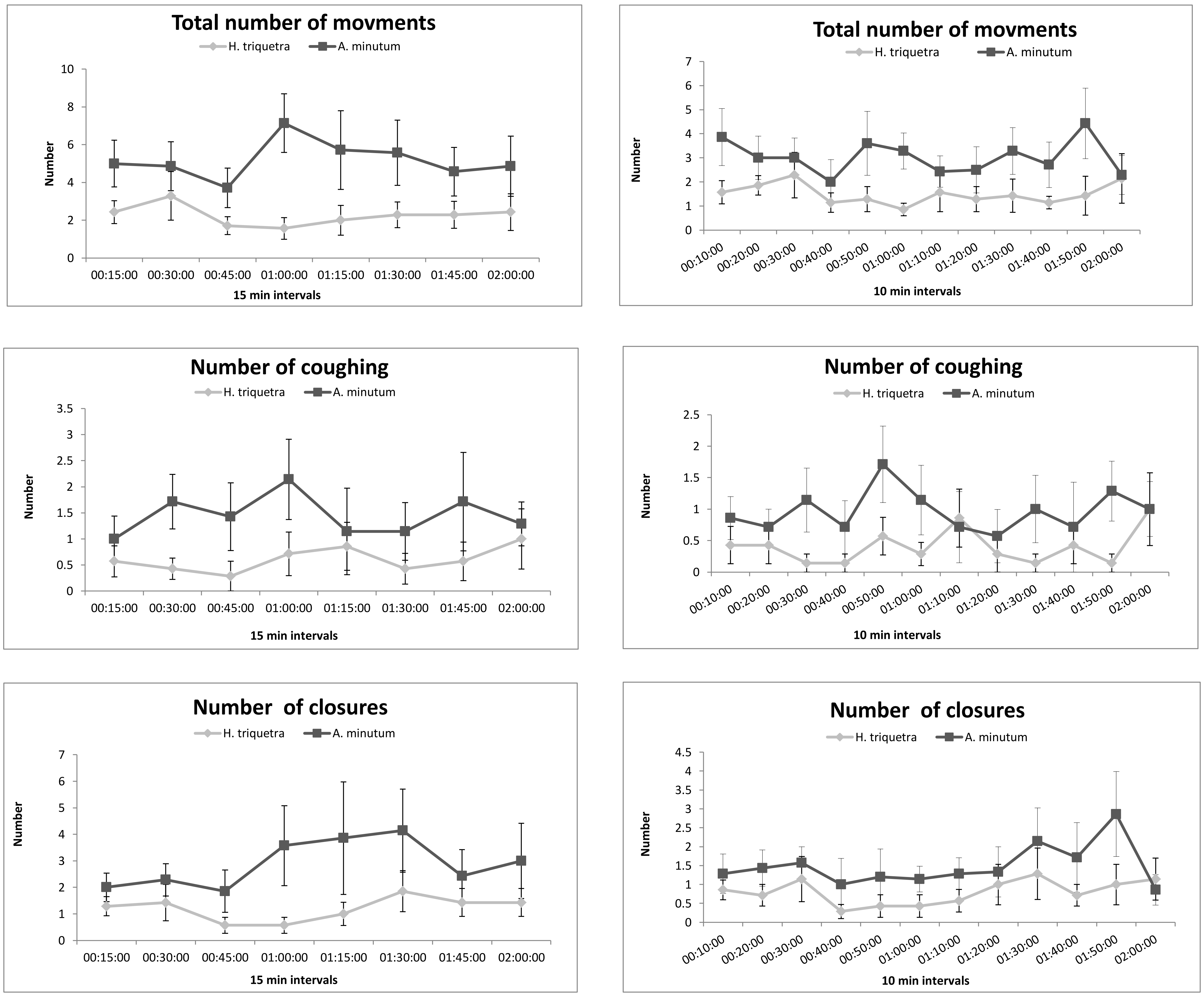

Supplement: S1 Fig — Scallop valve activity over the 2 hours of recording in response to exposition of Heterocapsa triquetra or toxic Alexandrium minutum. The examples here are under concentration expositions of 500 000 cell/L. (PDF) [file pone.0160935.s001.pdf]
