# Supplementary material for: Short-Term Behavioural Responses of the Great Scallop Pecten maximus Exposed to the Toxic Alga Alexandrium minutum Measured by Accelerometry and Passive Acoustics
Source: PLoS One. 2016 Aug 10;11(8):e0160935. doi: 10.1371/journal.pone.0160935 (PMC4980006; doi:10.1371/journal.pone.0160935)
Supplement: S1 Table — Summary of detected movement number found by both accelerometer and acoustics methods for each recording. (PDF) [file pone.0160935.s003.pdf]

Short-term behavioural responses of the great scallop *Pecten maximu* s exposed to the toxic alga *Alexandrium minutum* measured by accelerometry and passive acoustics

Laura Coquereau [a,*], Aurélie Jolivet [a,b], Hélène Hégaret [a], Laurent Chauvaud [a]

[a] *Université de Bretagne Occidentale, Institut Universitaire Européen de la Mer, Rue Dumont D'Urville, 29280 Plouzané, France*
[b] *TBM environnement/Somme, 115 rue Claude Chappe, Technopole Brest Iroise, F-29280 Plouzané, France*

Table 1 Summary of detected movement number found by both accelerometer and acoustics methods for each recording

Scallop	Diet	Concentration (cell/L)	Total number of movments		Number of coughing		Number of displacement		Number of closure		Number of swimming	
			Accelerometer	Acoustics	Accelerometer	Acoustics	Accelerometer	Acoustics	Accelerometer	Acoustics	Accelerometer	Acoustics
Coquille 1	*H. triquetra*	5 000	36	10	12	5	1	1	23	4	0	0
	A. minutum	5 000	24	8	18	7	0	0	6	1	0	0
Coquille 2	*H. triquetra*	5 000	14	4	3	1	0	0	11	3	0	0
	A. minutum	5 000	23	9	12	6	1	1	10	2	0	0
Coquille 3	*H. triquetra*	5 000	68	36	48	26	5	5	13	3	2	2
	A. minutum	5 000	40	11	6	3	0	0	34	8	0	0
Coquille 4	*H. triquetra*	5 000	9	2	3	2	0	0	6	0	0	0
	A. minutum	5 000	5	1	4	2	0	0	1	0	0	0
Coquille 5	*H. triquetra*	5 000	19	9	5	4	2	2	12	3	0	0
	A. minutum	5 000	22	9	5	3	1	1	16	5	0	0
Coquille 6	*H. triquetra*	5 000	30	11	6	4	4	4	20	3	0	0
	A. minutum	5 000	27	14	7	4	4	4	12	2	4	4
Coquille 7	*H. triquetra*	5 000	33	20	3	1	18	18	12	1	0	0
	A. minutum	5 000	44	25	10	7	15	15	17	1	2	2
Coquille 8	*H. triquetra*	5 000	9	8	3	3	3	3	3	2	0	0
	A. minutum	5 000	12	11	3	2	8	8	1	1	0	0
Coquille 9	*H. triquetra*	5 000	16	7	5	4	2	2	9	1	0	0
	A. minutum	5 000	15	7	2	2	2	2	9	1	2	2
Coquille 10	*H. triquetra*	10 000	36	12	9	5	2	2	25	5	0	0
	A. minutum	10 000	23	13	10	7	4	4	9	2	0	0
Coquille 11	*H. triquetra*	10 000	59	20	6	4	0	0	53	16	0	0
	A. minutum	10 000	41	15	5	4	0	0	36	11	0	0
Coquille 12	*H. triquetra*	10 000	10	3	3	1	0	0	7	2	0	0
	A. minutum	10 000	25	8	5	3	0	0	20	5	0	0
Coquille 13	*H. triquetra*	10 000	62	33	13	5	19	19	24	3	6	6
	A. minutum	10 000	24	13	1	1	7	7	10	0	6	6
Coquille 14	*H. triquetra*	10 000	9	2	1	1	0	0	8	1	0	0
	A. minutum	10 000	26	0	2	0	0	0	24	0	0	0
Coquille 15	*H. triquetra*	10 000	19	4	6	2	1	1	12	1	0	0
	A. minutum	10 000	10	6	4	3	0	0	6	3	0	0
Coquille 16	*H. triquetra*	10 000	31	6	8	2	1	1	20	1	2	2
	A. minutum	10 000	8	3	5	3	0	0	3	0	0	0
Coquille 17	*H. triquetra*	10 000	19	13	3	3	9	9	7	1	0	0
	A. minutum	10 000	42	24	5	2	19	19	18	3	0	0
Coquille 18	*H. triquetra*	10 000	52	30	8	4	22	22	18	0	4	4
	A. minutum	10 000	123	82	8	3	75	75	30	0	10	10
Coquille 19	*H. triquetra*	500 000	12	3	2	1	0	0	10	2	0	0
	A. minutum	500 000	18	5	6	3	0	0	12	2	0	0
Coquille 20	*H. triquetra*	500 000	26	7	6	4	0	0	20	3	0	0
	A. minutum	500 000	61	17	15	6	0	0	46	11	0	0
Coquille 21	*H. triquetra*	500 000	18	8	0	0	2	2	16	4	0	0
	A. minutum	500 000	22	10	1	0	2	2	15	4	4	4
Coquille 22	*H. triquetra*	500 000	21	7	14	6	0	0	7	1	0	0
	A. minutum	500 000	51	11	39	11	1	1	11	2	0	0
Coquille 23	*H. triquetra*	500 000	10	7	2	0	8	8	0	0	0	0
	A. minutum	500 000	7	7	3	3	3	3	0	0	1	1
Coquille 24	*H. triquetra*	500 000	21	9	7	3	8	8	6	1	0	0
	A. minutum	500 000	34	10	9	6	0	0	25	4	0	0
Coquille 25	*H. triquetra*	500 000	12	3	2	1	1	1	9	1	0	0
	A. minutum	500 000	58	40	8	4	33	33	13	1	4	4
Coquille 26	*H. triquetra*	500 000	18	14	5	2	12	12	1	0	0	0
	A. minutum	500 000	13	11	4	3	5	5	2	1	2	2
Coquille 27	*H. triquetra*	500 000	22	17	3	3	14	14	5	0	0	0
	A. minutum	500 000	64	19	7	4	8	8	49	7	0	0
